# Structural analyses of FERM domain-mediated membrane localization of FARP1

**DOI:** 10.1038/s41598-018-28692-4

**Published:** 2018-07-11

**Authors:** Yi-Chun Kuo, Xiaojing He, Andrew J. Coleman, Yu-Ju Chen, Pranathi Dasari, Jen Liou, Thomas Biederer, Xuewu Zhang

**Affiliations:** 1 Department of Pharmacology, University of Texas Southwestern Medical Center, Dallas, TX 75390 USA; 2 College of Life Science and Technology, Huazhong University of Science and Technology, Wuhan, China; 3 Department of Neuroscience, Tufts University School of Medicine, Boston, MA 02111 USA; 4 Department of Physiology, University of Texas Southwestern Medical Center, Dallas, TX 75390 USA

## Abstract

FARP1 is a multi-domain protein that is involved in regulating neuronal development through interacting with cell surface proteins such as class A Plexins and SynCAM 1. The N-terminal FERM domain in FARP1 is known to both promote membrane localization and mediate these protein interactions, for which the underlying molecular mechanisms remain unclear. Here we determined the crystal structures of the FERM domain of FARP1 from zebrafish, and those of FARP2 (a close homolog of FARP1) from mouse and zebrafish. These FERM domains adopt the three-leaved clover fold that is typical of all FERM domains. Our structures reveal a positively charged surface patch that is highly conserved in the FERM domain of FARP1 and FARP2. *In vitro* lipid-binding experiments showed that the FARP1 FERM domain binds specifically to several types of phospholipid, which is dependent on the positively charged surface patch. We further determined through cell-based analyses that this surface patch on the FERM domain underlies the localization of FARP1 to the plasma membrane, and that FERM domain interactions recruit it to postsynaptic sites in neurons.

## Introduction

FARP1 (FERM, RhoGEF and pleckstrin domain-containing protein 1) and its close homolog FARP2 were identified as guanine nucleotide exchange factors (GEFs) for RhoGTPases that play regulatory roles in neuronal development^1–4^. FARP1 has been shown to interact directly with the neuronal guidance receptors PlexinA1 and PlexinA4 and regulate signaling^5,6^. Activation of the Plexin receptor by its semaphorin ligand leads to signaling that drives morphological changes of neuronal axons and dendrites^7^. FARP1 is enriched in dendrites of lateral motor column neurons, where it serves as an effector of PlexinA4 to promote dendritic growth^5^. More recently, FARP1 has been shown to regulate synapse number and dendritic spine morphology^6,8^. This function of FARP1 is mediated, at least in part, by a direct interaction with the synaptogenic adhesion molecule SynCAM 1^8^. FARP2 also interacts with PlexinA family members, contributing to both Plexin-mediated repulsive axon guidance and dendritic development^9,10^. Recently, FARP2 has been found to be involved in Plexin-mediated regulation of bone homeostasis^11,12^.

**Figure 1:**
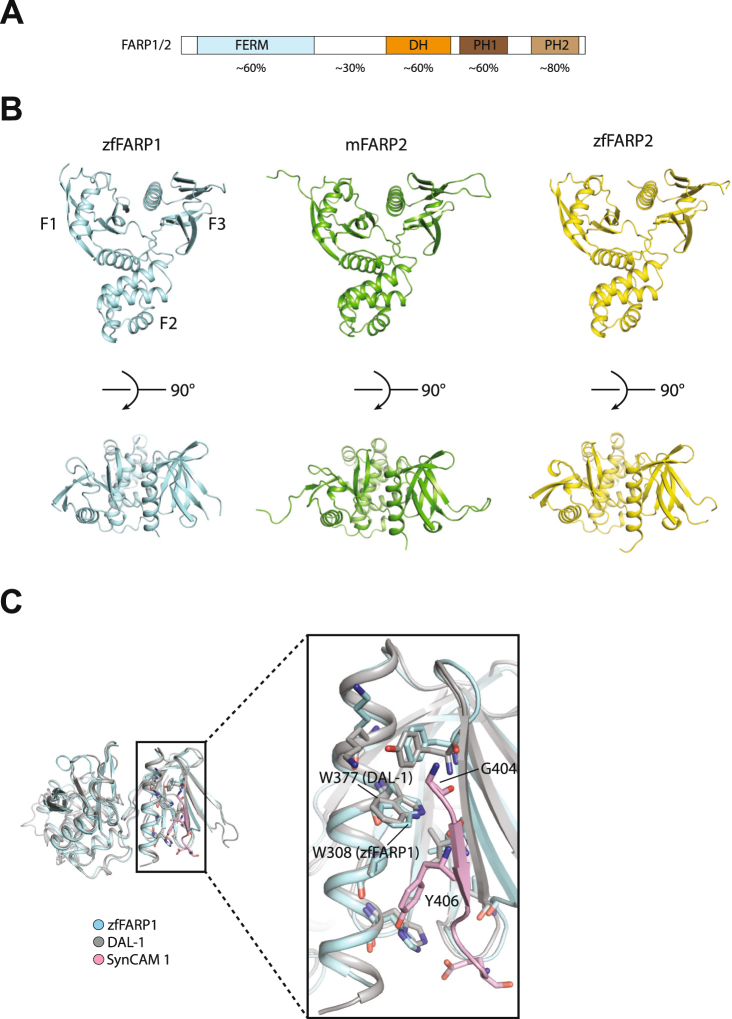
Structures of the FERM domains from zfFARP1, mFARP2 and zfFARP2. (**A**) Domain architecture of FARP1/2. Domain-wise sequence identity between zfFARP1 and zfFARP2 are shown at the bottom. (**B**) Overview of the FERM domain structures of zfFARP1, mFARP2 and zfFARP2. (**C**) Superimposition of the zfFARP1 FERM domain with DAL-1 in complex the C-terminal tail of SynCAM 1 (PDB ID: 3BIN).

FARP1 and FARP2 share a conserved domain architecture, containing a N-terminal 4.1, ezrin, radixin and moesin (FERM) domain, which is followed by a long linker (~200 residues) that connects to a Dbl-homology (DH) domain and two pleckstrin homology (PH) domains (Fig. 1A). The two proteins show high levels of sequence identity except for the non-conserved linker between the FERM and DH domains (Fig. 1A). The DH-PH tandem is a canonical feature of the Dbl-family GEFs for RhoGTPases^13^. Crystal structures of the DH-PH-PH domains of both FARP1 and FARP2 show an autoinhibited conformation where the RhoGTPase-binding site in the DH and the first PH (PH1) domain is blocked by the second PH domain (PH2) as well as several other structural elements in the protein^14^. Therefore, FARP1 and FARP2 cannot act as GEFs for RhoGTPases unless the autoinhibition is released through a conformational change. Alternatively, it has been suggested that FARP1 and FARP2 may be “pseudo-GEFs”, which have lost their GEF activity but regulate RhoGTPases through an indirect mechanism^14^.

The FERM domain in FARP1 mediates interactions with the intracellular sequences of the transmembrane proteins PlexinA4 and SynCAM 1^5,8^. The molecular details of these interactions are unknown due to lack of structural analyses. In addition to membrane proteins, many FERM domains can interact with phospholipids, which targets them to lipid membrane and facilitates their binding to cell surface proteins^15^. It is unclear whether the FERM domains in FARP1 and FARP2 have a similar ability to bind lipid membranes. To address these questions, we determined the crystal structures of the FERM domain of FARP1 from zebrafish as well as those of FARP2 from mouse and zebrafish. These structures and the associated biochemical and cell-biological analyses provide mechanistic insights into the interactions of these FERM domains with the plasma membrane and cell surface receptors, supporting FERM domain roles in the synaptic recruitment of FARP1 in neurons.

## Results

### Crystal structures of the FERM domains from FARP1 and FARP2

We determined crystal structures of the FERM domains of zebrafish FARP1 (zfFARP1), mouse FARP2 (mFARP2) and zebrafish FARP2 (zfFARP2) (Fig. 1B and Table 1). As expected from their high degree of sequence identity (>60%), the structures of these FERM domains are very similar to one another, with root mean square deviations within 1.0 Å. There are three subdomains (F1, F2 and F3) that assemble into a three-leaved clover shape (Fig. 1B), characteristic of all FERM domains^15^. The F1 subdomain exhibits a ubiquitin-like fold and consists of a five-strand β sheet capped by an α-helix. The structure of the F2 subdomain is constituted entirely with α-helices and structurally similar to acyl-CoA-binding proteins. The F3 subdomain is a β sandwich capped by a C-terminal α-helix, similar to PH and PTB (phospho-tyrosine binding) domains.

**Table 1:** Data collection and structure refinement statistics.

	mFARP2	zfFARP2	zfFARP1
**Data collection**			
Space group	P 2_1_ 2_1_ 2_1_	P 2_1_ 2_1_ 2_1_	P 2_1_ 2_1_ 2_1_
**Cell dimensions**			
a, b, c (Å)	39.42, 72.76, 94.23	35.38, 82.72, 96.02	56.03, 59.44, 97.46
α, β, γ (°)	90.00, 90.00, 90.00	90.00, 90.00, 90.00	90.00, 90.00, 90.00
Resolution (Å)	36.38-1.55(1.61-1.55)*	32.53-2.00(2.07-2.00)	40.77-2.99(3.10-2.99)
R_sym_ (%)	4.7(72.0)	8.3(61.4)	12.4(87.5)
R_pim_(%)	2.2(35.8)	3.3(32.5)	3.8(26.2)
I/σ	34.02(1.89)	22.53(2.20)	12.75(1.67)
CC_1/2_^#^	0.779	0.760	0.851
Completeness (%)	99.7(97.8)	98.60(88.07)	99.34(96.80)
Redundancy	5.4(4.8)	7.2(4.2)	11.6(11.9)
**Refinement**			
Resolution (Å)	36.38-1.55(1.61-1.55)	32.53-2.00(2.07-2.00)	40.77-2.99(3.10-2.99)
No. reflections	43891	19389	6910
R_work_/R_free_ (%)	19.7(25.6)/22.4(27.1)	18.3 (27.1)/23.9(35.1)	22.4(30.8)/28.3(35.5)
**No. atoms**			
Protein	2383	2296	2232
Ligand/ion	0	0	0
Water	290	145	0
**B-factors**			
Protein	22.10	43.50	81.20
**Ligand/ion**			
Water	32.30	46.10	—
**R.m.s deviations**			
Bond lengths (Å)	0.006	0.007	0.004
Bond angles (°)	1.03	0.98	0.91
**Ramanchandran plot**			
Favored (%)	98	99	96
Allowed (%)	2	1	3.63
Disallowed (%)	0	0	0.37

*Numbers in parenthesis are for the highest resolution shell.

^#^CC_1/2_ values shown are for the highest resolution shell.

Many FERM domains bind the cytoplasmic tail of cell surface proteins. One common binding site on the FERM domains is the surface groove formed between the last helix and the β5 strand in the F3 subdomain^15^. The cytoplasmic tail of cell surface proteins adopts an extended β-strand conformation to pack against β5 in F3 of the FERM domain. The FERM domains in FARP1 and FARP2 have been proposed to use this binding mode to interact with SynCAM 1 and class A Plexins, respectively^8,10^. A crystal structure of the C-terminal tail of SynCAM 1 bound to the FERM domain of DAL-1 (PDB ID: 3BIN) shows the detailed interactions in this mode (Fig. 1C)^16^. Gly404 in SynCAM 1 packs closely with Trp377 in the last helix of the FERM domain. Tyr406 in SynCAM 1 is buried in the hydrophobic groove between the last helix and β5 in the FERM domain. This interaction mode explains the common “GXY” (“X” denotes any residue) motif found in cytoplasmic tails of cell surface proteins that bind FERM domains. While the FERM domains in FARP1 and DAL-1 only share ~40% sequence identity, the residues in the binding groove of these two FERM domains are nearly identical as shown by the structural superimposition (Fig. 1C), strongly suggesting that the interaction between FARP1 and SynCAM 1 uses the same binding mode.

### Conserved positively charged surface patch on the FERM domain in FARP1 and FARP2

**Figure 2:**
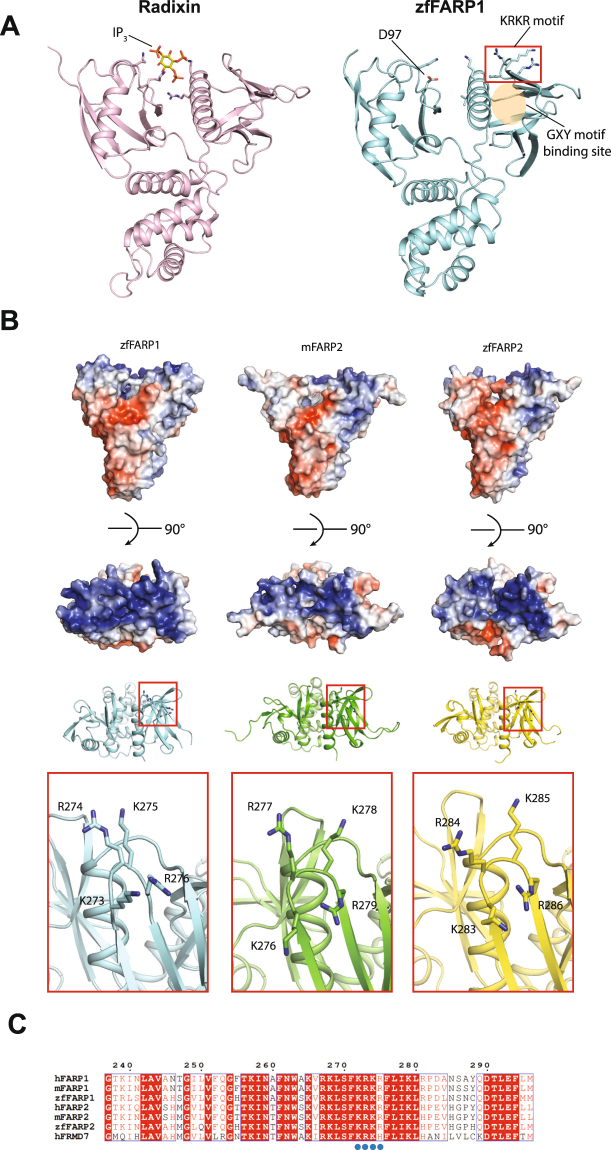
Potential membrane binding surface on the FERM domain of FARP1 and FARP2. (**A**) Comparison of the FERM domains of Radixin and zfFARP1. IP_3_ and the positively charged residues in Radixin contributing to IP_3_ binding are shown in the left panel. The corresponding residues in the zfFARP1 FERM domain are shown in the right panel, with the negatively charged residue (Asp97) highlighted. The KRKR motif in the zfFARP1 is highlighted by the red rectangle. The oval denotes the GXY motif binding site. (**B**) Surface electrostatic potential (upper panels) and the KRKR motifs (lower panels) in the FERM domains of zfFARP1, mFARP2 and zfFARP2. The red-to-blue color gradient indicates the range of electrostatic potential (−5 kT/e to 5 kT/e). The rectangles denote the KRKR motif. (**C**) Sequence alignment showing the conservation of the KRKR motif in FARP1/2 from different species and in human FRMD7.

FERM domains are known to interact with phospholipids such as phosphatidylinositol, which is one of the mechanisms by which FERM-containing proteins localize to lipid membrane^17^. The crystal structure of the FERM domain of Radixin in complex with inositol-(1,4,5)-trisphosphate (IP_3_) (PDB ID: 1GC6) shows that the phosphatidylinositol binding site is located at the junction between subdomains F1 and F3, where several positively charged residues interact with the phosphate groups on IP_3_ (Fig. 2A)^18^. The corresponding site in the FERM domains of FARP1 and FARP2 contains a negatively charged residue (Asp97, Glu100 and Asp107 in zfFARP1, mFARP2 and zfFARP2, respectively), suggesting that it cannot serve as a phosphatidylinositol binding site in these FERM domains (Fig. 2A). The FERM domains in FARP1 and FARP2 however do contain many positively charged residues. Overall, the surface of these FERM domains show highly polarized surface electrostatic potential, with one side positively charged while the opposite side negatively charged (Fig. 2B). According to the binding mode between the FARP1 FERM and SynCAM 1 proposed above, the side of the FERM domain proximal to the membrane displays positive electrostatic potential (Fig. 2B). The positively charged surface on the FERM domain likely interacts with the negatively charged lipid membrane surface, thereby facilitating the membrane localization of FARP1 and its interaction with SynCAM 1. In particular, the FERM domains of FARP1 and FARP2 contain a Lys-Arg-Lys-Arg (KRKR) motif in the loop connecting strands β5 to β6 in the F3 subdomain, which is highly conserved in FARP1/2 from different species (Fig. 2B,C). A similar motif is also present in FRMD7 (FERM domain-containing protein 7), which has a FERM domain that is closely related to that in FARP1 and FARP2 (~60% sequence identity) (Fig. 2C)^19,20^. The KRKR motif constitutes a major part of the positively charged surface, and may serve as a lipid binding site for targeting FARP1/2 to the plasma membrane.

### FERM domain of FARP1 directly binds to phospholipids *in vitro*

The FERM domain of both FARP1 and FARP2 tended to precipitate during purification, which was alleviated with increased salt concentrations (higher than 500 mM NaCl) (See methods). These observations suggest that these FERM domains in solution may oligomerize through electrostatic interactions. Interestingly, both the FERM domains of mFARP2 and zfFARP2 form similar head-to-tail packing patterns in the crystal lattices, as a result of interactions between the positively charged and the negatively charged faces of the proteins (Fig. S1A). These packing patterns lead to a linear array of the FERM molecules, which may cause the oligomerization and precipitation of the proteins. The first arginine residue in the KRKR motif (Arg277) in the FERM domain of mFARP2 makes a salt bridge with Asp216 from the neighboring protomer in the linear array (Fig. S1B). An equivalent salt bridge (Arg284-Asp223) is also present in the structure of the FERM domain of zfFARP2 (Fig. S1C). The FERM domain of human FARP1 (hFARP1) with the corresponding aspartate residue (Asp212) mutated to asparagine (D212N) was much less prone to precipitation. We therefore used the D212N mutant to test the interaction between the FERM domain of hFARP1 and phospholipids. We used a protein-lipid overlay assay that is based on binding of proteins to membrane strips spotted with an array of common phospholipids^21^, in which precipitation of the protein often leads to high background and false positive results.

**Figure 3:**
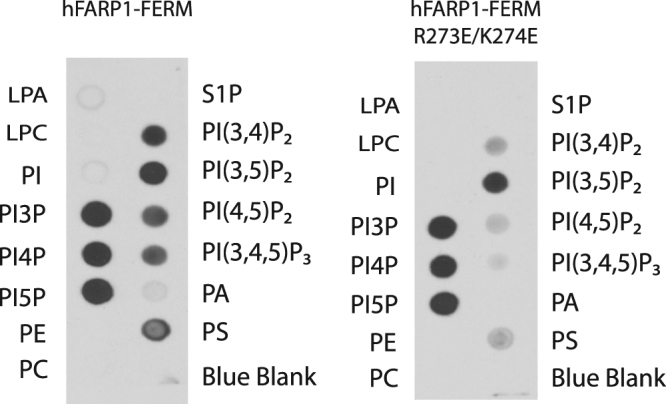
Protein-lipid overlay assay for the interactions of the hFARP1 FERM with phospholipids. LPA: Lysophosphatidic Acid, LPC: Lysophosphocholine, PI: Phosphatidylinositol, PI3P: Phosphatidylinositol(3)phosphate, PI4P: Phosphatidylinositol(4)phosphate, PI5P: Phosphatidylinositol(5)phosphate, PE: Phosphatidylethanolamine, PC: Phosphatidylcholine, S1P: Sphingosine-1-phosphate, PI(3,4)P_2_: Phosphatidylinositol(3,4)bisphosphate, PI(3,5)P_2_: Phosphatidylinositol(3,5)bisphosphate, PI(4,5)P_2_: Phosphatidylinositol(4,5)bisphosphate, PI(3,4,5)P_3_: Phosphatidylinositol(3,4,5) trisphosphate, PS: Phosphatidylserine, PA: Phosphatidic Acid. The figures were cropped from two single blots.

The results showed that the FERM domain of hFARP1 bound robustly to a variety of phospholipids, including phosphatidylinositol (PI)-3-phospate (PI3P), PI4P, PI5P, PI(3,4)P_2_, PI(3,5)P_2_, PI(4,5)P_2_, PI(3,4,5)P_3_, and Phosphatidylserine (PS), but not Lysophosphatidic Acid (LPA), Lysophosphocholine (LPC), PI, Phosphatidylethanolamine (PE), Phosphatidylcholine (PC), Sphingosine-1-phosphate (S1P), or Phosphatidic Acid (PA) (Fig. 3). The FERM domain bearing a double mutation in the KRKR motif (R273E/K274E) showed dramatically deceased binding to PI(3,4)P_2_, PI(4,5)P_2_, PI(3,4,5)P_3_, and PS. In contrast, the binding to PI3P, PI4P, PI5P and PI(3,5)P2 was not substantially affected by the mutations. These results together suggest that the FARP1 FERM interacts specifically with certain types of phospholipid, and the KRKR motif is important for some of these interactions. Of particular note, the KRKR motif is essential for binding to PI(4,5)P_2_, the most abundant type of phosphatidylinositol on the plasma membrane^22^.

### FERM domain targets FARP1 to the plasma membrane

**Figure 4:**
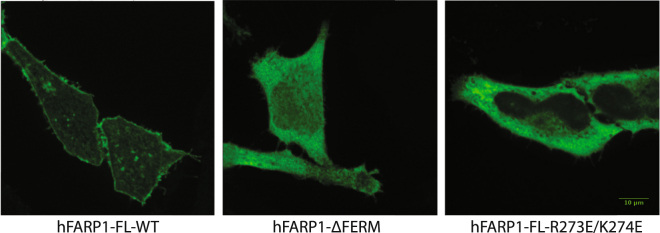
FERM-dependent membrane localization of hFARP1. Middle sections of representative confocal images of HeLa cells expressing various constructs of hFARP1 are shown. hFARP1-FL-WT, full-length hFARP1; hFARP1-ΔFERM, hFARP1 with FERM domain truncated; hFARP1-FL-R273E/K274E, full-length hFARP1 with the R273E/K274E double mutations in the KRKR motif.

The subcellular localization of FARP1 in cells has been analyzed by previous studies. Two studies showed that FARP1 was present both in the cytosol and at the plasma membrane, but localized predominantly to the membrane in the presence of SynCAM 1 or Plexin^6,8^. Another study showed that FARP1 was constitutively localized to the plasma membrane^23^. To further analyze the membrane localization of FARP1 and the role of the FERM domain in this process, we used immunofluorescence to determine the subcellular localization of FLAG-tagged full-length human FARP1 expressed in HeLa cells with an anti-FLAG antibody. Confocal microscopy images showed that full-length FARP1 is localized predominantly on the plasma membrane. In contrast, FARP1 with the FERM domain truncated distributed evenly in the cytosol, suggesting that the FERM domain is essential for the membrane targeting of FARP1. The double mutation (R273E/K274E) of the KRKR motif in the FERM domain also abolished the membrane localization of FARP1 (Fig. 4), supporting the notion that this motif contributes to plasma membrane localization through binding to PI(4,5)P_2_.

### Postsynaptic localization of FARP1 in neurons involves FERM domain interactions

**Figure 5:**
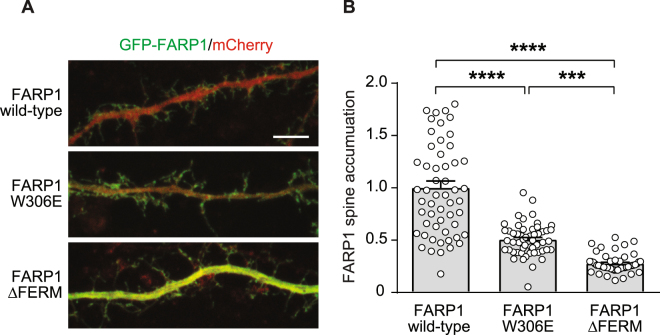
FERM domain targets FARP1 to dendritic spines. (**A**) Representative confocal images of GFP-tagged FARP1 wild-type, W306E and ΔFERM in mCherry transfected dissociated hippocampal neurons at 21 div. Scale bar, 5 μm. (**B**) Relative levels of spine enrichment of GFP-tagged FARP1 variants. Spine enrichment of GFP signal was normalized to spine area and calculated as the fluorescence intensity of (GFP/mCherry) spine/(GFP/mCherry) dendrite. WT (1.00 ± 0.07), n = 55 spines from 10 neurons); W306E (0.51 ± 0.02), n = 53/10; ΔFERM (0.28 ± 0.01), n = 50/10. ***p < 0.001, ****p < 0.0001. ANOVA followed by Tukey’s multiple comparison test.

FARP1 and SynCAM 1 co-localize in neuronal dendritic spines, the postsynaptic sites onto which most excitatory synapses are formed^8^. To analyze roles of FERM domain interactions in the spine localization of FARP1, we developed GFP-tagged wild-type and FERM domain mutants. We mutated Trp306 in human FARP1 to glutamate (W306E) to disrupt FARP1-SynCAM 1 interactions. According to the FERM/SynCAM 1 binding mode discussed above, Trp306 (corresponding to Trp308 in zebrafish FARP1) is important for interacting with the “GXY” motif in SynCAM 1 (Fig. 1C). We also generated a FERM domain deletion mutant (ΔFERM) that lacks the N-terminal sequence including the FERM adjacent region. We validated expression of these mutants after heterologous expression in HEK293 cells by immunoblotting (Fig. S2). GFP-tagged wild-type FARP1 and its mutants were transfected into dissociated hippocampal neurons along with soluble mCherry, which served as a cell fill to outline dendrites and their spine protrusions (Fig. 5A). As previously shown, wild-type FARP1 exhibited at 21 days *in vitro* (div) a pronounced enrichment in dendritic protrusions^8^. We compared the spine enrichment of wild-type FARP1 and its FERM domain variants by measuring the ratio of GFP signal in spines versus the adjacent dendritic shaft, normalized by the mCherry signal. The W306E mutant showed a 50 ± 5.9% reduction in spine enrichment compared to wild-type FARP1 (Fig. 5B). The FARP1 ΔFERM showed an even stronger reduction (72 ± 6.0%) in spine accumulation than wild-type FARP1. The spine localization of the FARP1 ΔFERM was significantly lower than measured for the W306E mutant. These results support that FERM domain interactions contribute importantly to the dendritic spine enrichment of FARP1 in developing neurons.

Together our structural analyses, *in vitro* lipid binding assay and cell-based localization assays support that the FERM domain uses the positively charged surface, particularly the KRKR motif, to interact with phospholipids and target FARP1 to the plasma membrane, thereby increasing its potential to interact with cell surface proteins such as SynCAM 1 and Plexin.

## Discussion

Our structural and cell biological analyses of the FERM domains of FARP1 and FARP2 suggest that they interact with their binding partners such as SynCAM 1 on the cell surface through the canonical binding mode that is used by many FERM domains. Class A Plexins have also been shown to interact with the FERM domains of FARP1 and FARP2. However, the cytoplasmic region of Plexins does not contain a “GXY” motif as in SynCAM 1 that is critical for binding to the FERM domain. Understanding the binding mode between the FERM domain of FARP1/2 and Plexins requires further investigation.

**Figure 6:**
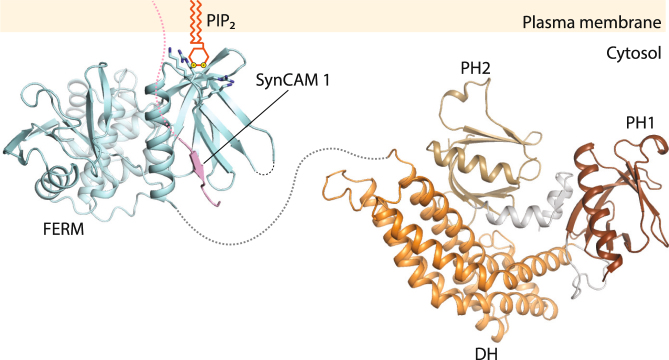
Model of FARP1 membrane localization and interaction with the cytoplasmic tail of SynCAM 1.

We show that the positively charged surface patch containing the KRKR motif in the FARP1 FERM domain is a novel binding site for phospholipids that is responsible for targeting FARP1 to the plasma membrane. This surface patch is present in both FARP1 and FARP2 from different species as well as FRMD7 but not generally conserved in all FERM domains, suggesting that this is a specialized membrane targeting mechanism used by this sub-family of FERM containing proteins. The binding sites for proteins and lipids in the FERM domains of FARP1 and FARP2 are arranged in such a manner that both binding events can engage simultaneously and may be synergistic in recruiting FARP1 and FARP2 to the cell surface for transducing signal of transmembrane proteins (Fig. 6). This model agrees with our finding that the deletion of the FERM domain impairs postsynaptic recruitment of FARP1 even more than a point mutation predicted to impair interactions with GXY motifs of membrane proteins.

Both FARP1 and FARP2 contain two PH domains, which also contain potential binding sites for phospholipids^14^. Surprisingly, our cell-based assays showed that the FERM domain in FARP1 is necessary and sufficient for its targeting to the plasma membrane. The binding of the PH domains to phospholipids may be weak and cannot localize FARP1 to the membrane on their own, or they only bind certain types of phosphatidylinositol that are not present on the plasma membrane under the conditions in our experiments.

The linear packing of the FERM domains of mFARP2 and zfFARP2 in our crystal structures suggest a potential mechanism for the oligomerization of these FERM domains. The KRKR motif in the FERM domains responsible for phospholipid binding is buried in the oligomerization interface. We speculate that the oligomerization, if occurs in the cell, could be a mechanism for regulating membrane targeting of FARP1 and FARP2 (Fig. S1).

## Materials and Methods

### Protein Expression and Purification

The coding regions of the FERM domain of hFARP1 (residues 36–329), zfFARP1 (residues 38–322), mFARP2 (residues 38–324), and zfFARP2 (residues 49–378), were cloned into a modified pET-28(a) vector that encodes an N-terminal His_6_-tag followed by a SUMO-tag, and a recognition site for Ulp SUMO protease. The cDNAs of the FERM domains of hFARP1 and mFARP2 were from OpenBiosystem. The cDNAs of FERM domains of zfFARP1 and zfFARP2 were synthesized by GenScript with codon optimization. The D212N mutation was introduced to hFARP1 FERM through QuikChange. The plasmids were transformed into the *E. coli* strain BL21(DE3) for protein expression, induced by 0.2 mM IPTG at 16 °C overnight. Bacterial lysates were cleared by centrifugation and the supernatants were passed through a 1 mL HisTrap column (GE Healthcare) and washed with Buffer A containing 10 mM Tris (pH 8.0), 1 M NaCl, 5% glycerol (v/v), 20 mM Imidazole, and 3 mM β-mercaptoethanol. On-column digestion of the His_6_-SUMO-tag was carried out by injecting recombinant SUMO protease in Buffer A to the HisTrap column and incubating at 4 °C overnight. The FERM domain proteins were eluted with Buffer A and further purified by a Superdex 200 HiLoad 16/60 gel filtration column (GE healthcare) equilibrated with Buffer B containing 20 mM Tris (pH 8.0), 500 mM NaCl, 10% glycerol (v/v), and 2 mM DTT. Purified proteins were concentrated and stored at −80 °C. These FERM domains needed NaCl at 500 mM or higher concentrations to remain soluble. For the hFARP1 FERM domain, the N-terminal His_6_-SUMO tag was uncleaved and used for Western blotting detection in the protein-lipid overlay assay.

### Crystallization and Structure Determination

Initial crystallization trials were set up in sitting-drop 96-well plates at the 1:1 volume ratio between the protein and crystallization solutions, at 4 °C for the zfFARP1 FERM domain and at 20 °C for the FERM domains of mFARP2 and zfFARP2. High quality crystals of the mFARP2 FERM for X-ray data collection were obtained in 0.1 M sodium acetate trihydrate pH 7.0 and 12% w/v Polyethylene glycol (PEG) 3350 (w/v). Crystals of the zfFARP2 FERM domain were grown in 0.2 M potassium sodium tartrate tetrahydrate and 20% PEG3350 (w/v). The zfFARP1 FERM domain was crystallized in 0.1 M Bicine pH 9.0 and 16% (+/−)-2-Methyl-2,4-pentanediol (MPD) (v/v). Crystals were cryo-protected using the crystallization solution supplemented with 20–30% glycerol and flash cooled in liquid nitrogen. Diffraction data were collected at −173 °C on beamline 19ID at the Advanced Photon Source (Argonne National Laboratory). Data were indexed, integrated and scaled by using HKL2000^24^. The structure of the mFARP2 FERM domain was solved by molecular replacement using the Phaser program in the Phenix package with the structure of the DAL-1 FERM domain (PDB ID: 2HE7) as the search model^25,26^. The structure of the mFARP2 FERM was then used as the search model to solve the structures of the FERM domains of zfFARP1 and zfFARP2. Iterative model building and structure refinement were performed by using the programs Coot and Phenix, respectively^25,27^. Statistics of data collection and refinement are listed in Table 1. Molecular structure figures were rendered by the program PyMOL (the PyMOL Molecular Graphics System, Schrödinger). Sequences were aligned by using MAFFT with default settings and BLOSUM62 as the scoring matrix for amino acid sequences^28^ and rendered with ESPript^29^.

### Immunostaining and confocal imaging

HeLa cells purchased from American Type Culture Collection were cultured in DMEM supplemented with 10% fetal bovine serum (HyClone) and 1x penicillin/streptomycin solution. Cells were plated on 8-well Lab-Tek chambered cover glass (Nunc) at a low density the day before transfection. 100 ng of C-terminal Flag-tagged hFARP1 WT, FERM deleted, and R273E/K274E in the pCDNA3.1(+) vector (Invitrogen) were transfected to HeLa cells using Fugene HD (Promega). 12–14 hrs after transfection, cells were fixed using 4% formaldehyde (Thermo scientific), immunostained with an anti-FLAG primary antibody (Sigma, Cat#F1804) and an Alexa-488 conjugated secondary antibody (Invitrogen, Cat#A11029). Cells were washed with PBS and imaged in PBS at room temperature with a 60x objective on a spinning-disc confocal system built around a Ti-E Perfect Focus microscope (Nikon) with an EM camera (c9100-13; Hamamatsu) controlled by Micro-Manager software^30^.

### Protein-lipid overlay assay

The protein-lipid overlay assay as described previously^21^ was used to examine the interaction between the hFARP1 FERM (D212N and D212N/R273E/K274E) and phospholipids. The N-terminal His_6_-SUMO tag in the proteins were used for detection by an anti-His_6_ tag antibody (Clontech, Cat# 631212) and an HRP-conjugated secondary antibody (MP Biomedicals, Cat# 0855565). PIP strips (P-6001) were purchased from Echelon.

### Immunoblotting

Proteins from cell lysates were subjected to immunoblotting using standard procedures (Fogel *et al*., 2007) and scanned with a FluorChem M Imaging System (Protein Simple, San Jose, CA). Primary antibodies used for immunoblotting were an anti-FARP1 antibody raised in guinea pig against the C-terminal peptide^8^ and a monoclonal antibody against GAPDH (Millipore, Cat# MAB374).

### Dendritic spine localization

Dissociated hippocampal cultures were prepared from rats at E18 as previously described (Salzberg *et al*., 2017). In brief, dissected hippocampi were incubated in 0.05% trypsin at 37 °C for 20 minutes and plated at a density of 60,000 cells per 12 mm coverslip coated with poly-l-lysine (Sigma P1274). Cells were incubated in a cell culture incubator maintained at 37 °C with 5.0% CO_2_. Cytosine arabinoside (Ara-c, Sigma C1768) was added at a final concentration of 2 μM at 2 days *in vitro* (DIV) to prevent glia cell overgrowth before the medium was replaced with Neurobasal without Ara-c at 4 days *in vitro* (div). Neurons were transfected at 7 div using Lipofectamine LTX and Plus Reagent (ThermoFisher). For neuronal transfections, the FARP1 cDNA was subcloned into a pCAG-BGH-pA and tagged with N-terminal GFP tag. The W306E mutation was introduced by PCR mutagenesis and the FARP1 ΔFERM mutant generated by PCR amplification of an open reading frame beginning at amino acid 441 in human FARP1. The mCherry vector has been previously described (Stagi *et al*., 2010). At 21 div coverslips with neurons were quickly washed two times with PBS, followed by fixation for 15 min with 4% PFA/4% sucrose in PBS at RT, washed 3 times with PBS, and mounted on slides Aqua-Mount mounting media (Thermo Scientific). Dendrites were imaged on a Leica SPE confocal microscope using a 63X objective with a 5x digital zoom. Z-stacks were collected at interval of 0.33 μm and maximum intensity projections analyzed. Images were analyzed with the experimenter blinded to the condition and fluorescence intensity was measured using ImageJ along line scans from dendritic protrusions into the adjacent dendritic shaft. Spine enrichment of GFP-tagged FARP1 and mutant constructs was calculated as fluorescence intensity of (GFP/mCherry) spine head/(GFP/mCherry) dendritic shaft and normalized to the wild-type condition.

### Data availability

The atomic coordinates and structure factors for the FERM domains of zfFARP1, mFARP2, and zfFARP2 have been deposited to the protein data bank, with the PDB access codes of 6D2Q, 6D2K, and 6D21, respectively.

## Electronic supplementary material

Supplementary Information
