# Supplementary material for: Structural analyses of FERM domain-mediated membrane localization of FARP1
Source: Sci Rep. 2018 Jul 11;8:10477. doi: 10.1038/s41598-018-28692-4 (PMC6041286; doi:10.1038/s41598-018-28692-4)
Supplement: Supplementary file 1 — Supplementary Information [file 41598_2018_28692_MOESM1_ESM.pdf]

Structural analyses of FERM domain-mediated membrane localization of FARP1

Yi-Chun Kuo[1,*], Xiaojing He[2,*], Andrew Coleman[3], Yu-Ju Chen[4], Pranathi Dasari[1], Jen Liou[4], Thomas Biederer[3] and Xuewu Zhang[1,#]

[1] Department of Pharmacology, University of Texas Southwestern Medical Center, Dallas, TX 75390

[2] College of Life Science and Technology, Huazhong University of Science and Technology, Wuhan, China

[3] Department of Neuroscience, Tufts University School of Medicine, Boston, MA 02111, USA

[4] Department of Physiology, University of Texas Southwestern Medical Center, Dallas, TX 75390

[*] Equal contributions

[#] Corresponding author: Xuewu Zhang (xuewu.zhang@utsouthwestern.edu)

Keywords: FARP1, FERM, SynCAM, plasma membrane

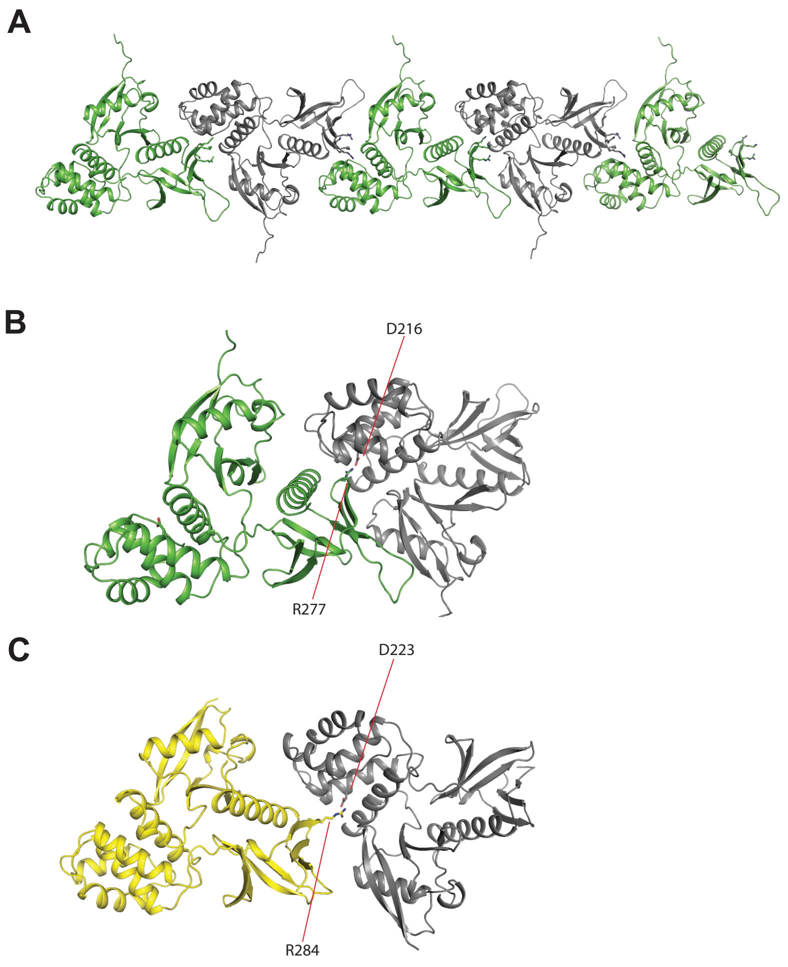

Figure S1. Head-to-tail oligomers in the crystal lattices of the FERM domains of mFARP2 and zfFARP2. (A) Head-to-tail oligomer in the mFARP2 FERM crystal lattice. The FERM domains are colored in alternating green and gray for clarity. (B) Salt bridge between Arg277 from one FERM domain and Asp216 from its neighbor in the mFARP2 FERM oligomer. (C) The equivalent salt bridge in the zfFARP2 FERM oligomer (between Arg284 and Asp223).

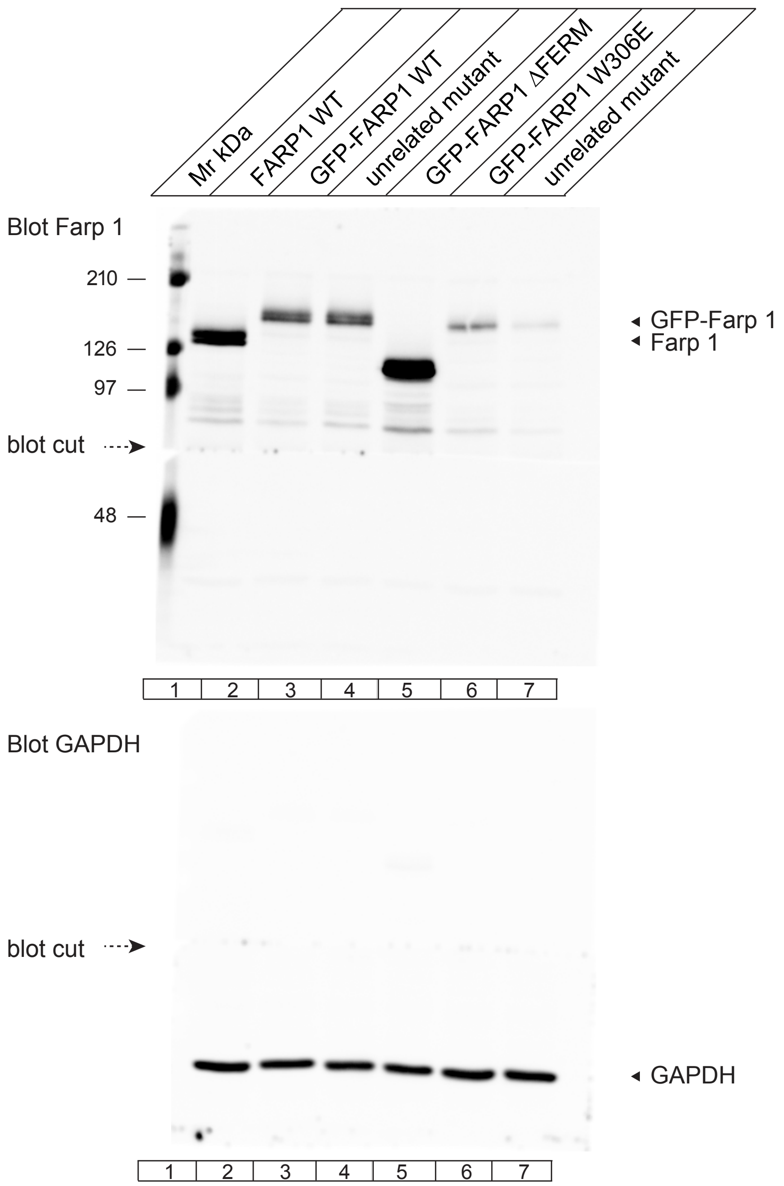

Figure S2. Immunoblots validating expression of FARP1 FERM domain mutants used for dendritic localization analysis. Uncropped immunoblots of total cell lysates from HEK293 cells transfected with GFP-tagged full-length wild-type (WT), W306E, and ΔFERM FARP1 mutants used for the dendritic localization in Figure 5. Top, a FARP1 antibody recognizing the C-terminus of the protein was used to validate the expression of these mutants at the expected molecular weights. Bottom, GAPDH served as a loading control. Additional mutants not described in this study were also included in this uncropped blot.
